# High CD8^+^tumor-infiltrating lymphocytes indicate severe exhaustion and poor prognosis in angioimmunoblastic T-cell lymphoma

**DOI:** 10.3389/fimmu.2023.1228004

**Published:** 2023-09-15

**Authors:** Qiqi Zhu, Yiming Yang, Xueqin Deng, Ningning Chao, Zihang Chen, Yunxia Ye, Wenyan Zhang, Weiping Liu, Sha Zhao

**Affiliations:** ^1^ Department of Pathology, West China Hospital, Sichuan University, Chengdu, China; ^2^ Department of Pathology, North Sichuan Medical College, Affiliated Hospital of North Sichuan Medical College, Nanchong, China; ^3^ Institute of Basic Medicine and Forensic Medicine, North Sichuan Medical College, Nanchong, China; ^4^ Institute of Respiratory Health, Frontiers Science Center for Disease-related Molecular Network, West China Hospital, West China School of Medicine, Sichuan University, Chengdu, China

**Keywords:** angioimmunoblastic T-cell lymphoma, CD8 + tumor-infiltrating lymphocytes, exhaustion, immune checkpoint, single-cell RNA sequencing, prognosis, therapy

## Abstract

**Background:**

Exhaustion of CD8^+^ tumor-infiltrating lymphocytes (TILs), characterized by the overexpression of immune checkpoints (IC), is a major impediment to anti-tumor immunity. However, the exhaustion status of CD8^+^TILs in angioimmunoblastic T cell lymphoma (AITL) remains unclear. Therefore, we aimed to elucidate the exhaustion status of CD8^+^TILs in AITL and its influence on prognosis.

**Methods:**

The correlation between CD8^+^TILs and IC expression in AITL was analyzed using single-cell RNA sequencing (n = 2), flow cytometry (n = 20), and RNA sequencing (n = 20). Biological changes related to CD8^+^TILs exhaustion at different cytotoxic T lymphocyte (CTL) levels (mean expression levels of CD8A, CD8B, GZMA, GZMB, and PRF1) in AITL were evaluated using RNA sequencing (n = 20) and further validated using the GEO dataset (n = 51). The impact of CD8 protein expression and CTL levels on patient prognosis was analyzed using flow cytometry and RNA sequencing, respectively.

**Results:**

Our findings demonstrated that the higher the infiltration of CD8^+^TILs, the higher was the proportion of exhausted CD8^+^TILs characterized by the overexpression of multiple IC. This was accompanied by extensive exhaustion-related biological changes, which suggested severe exhaustion in CD8^+^TILs and may be one of the main reasons for the poor prognosis of patients with high CD8^+^TILs and CTL.

**Conclusion:**

Our study comprehensively reveals the exhaustion status of CD8^+^TILs and their potential negative impact on AITL prognosis, which facilitates further mechanistic studies and is valuable for guiding immunotherapy strategies.

## 1 Introduction

Angioimmunoblastic T-cell lymphoma (AITL) is a common type of peripheral T-cell lymphoma (PTCL) accounting for 15–30% of PTCL (1). AITL originates from T-follicular helper cells and mainly occurs in elderly patients with a heterogeneous prognosis. Most clinical manifestations of AITL, including polyclonal hypergammaglobulinemia, circulating immune complexes, and cold agglutinin with hemolytic anemia, represent an impaired immune system (2). The AITL microenvironment is rich in multiple cell infiltrates, including tumor-infiltrating lymphocytes (TILs), macrophages, eosinophils, and mast cells, of which TILs account for a large proportion and play a critical role in AITL.

We evaluated the relationship between TILs and AITL prognosis using flow cytometry in our previous study, which was the largest cohort focusing on TILs in *de novo* AITL using fresh lymph node samples (3). Our results suggested that an increase in CD8^+^TILs, characterized by anti-tumor immunity impairment, was related to an inferior prognosis (3, 4). Notably, the impact of CD8^+^TILs on AITL was contrary to the results for most other tumors (5, 6); we thus hypothesized that CD8^+^TILs in AITL may be dysfunctional or exhausted. CD8^+^TILs play a central role in exerting effector functions, while CD8^+^TILs exhaustion, characterized by upregulated expression of immune checkpoints (IC), leads to a serious deficiency in anti-tumor responses. Therefore, in this study, we analyzed the correlations among CD8^+^TILs and expression of IC, exhaustion-related biological changes, and prognosis of patients with AITL, to elucidate the exhaustion status of CD8^+^TILs in AITL and to further explore its influence on AITL occurrence and development as well as to determine possible therapeutic targets.

## 2 Patients and methods

### 2.1 Patients

*De novo* AITL cases were identified from January 2015 to September 2021 at the Department of Pathology of West China Hospital, Sichuan University. Three expert hematopathologists (S. Z., W. Z., and W. L.) independently reviewed all candidate cases using WHO-classified diagnostic criteria for AITL (4th edition, 2008/Revised 4th edition, 2017) and provided unanimous diagnoses. Patients with recurrent or secondary AITL were excluded. Relevant clinical information was obtained by reviewing electronic medical records. Follow-up information was collected in December 2022 through telephone interviews or electronic medical record reviews. Survival time was calculated from the date of pathological diagnosis to the date of death or last follow-up. The study was approved by the Ethics Committee on Biomedical Research, West China Hospital of Sichuan University. The committee waived the requirement for informed consent as the data for the patients included in the study were retrospectively analyzed.

### 2.2 Single-cell RNA sequencing

Single-cell RNA sequencing (scRNA-seq) was performed at the Department of Respiratory and Critical Care Medicine, Frontiers Science Center for Disease-Related Molecular Networks, West China Hospital, Sichuan University, China. The scRNA-seq procedures included sample collection and processing, single-cell RNA library construction and sequencing, and scRNA-seq data processing and analysis. Details of scRNA-seq are provided in the **Supplementary Materials** and **Table 1**.

**Table 1:** The baseline of patients with AITL in scRNA-seq.

Patient	Gender	Age	Stage	PS 2-5	IPI 2-5	LDH>220 IU/L	Extranodal involvement	Treatment	Response to treatment	Clnical status
AITL-1	female	70	IIIA	+	+	–	–	CHOP+Chidamide	SD	alive, 10.5 months
AITL-2	male	57	IIIB	–	+	+	–	CHOP+Chidamide	PR	alive, 10.8 months

### 2.3 Flow cytometry

The expression of IC (PD-1, CTLA-4, TIM3, LAG3, and TIGIT) on CD8^+^TILs was evaluated in fresh lymph node samples from 20 patients with *de novo* AITL using flow cytometry (FCM) with the following panels (FITC/PE/PerCP-Cy5.5/PE-Cy7/APC/APC- Cy7/AmCyan): (1) -/PD-1/CD8/CD4/CTLA-4/CD45/CD3/CD19 and (2) TIM3/LAG3/CD8/CD4/TIGIT/CD45/CD3/CD19. The detailed procedures have been described in our previous study (7).

### 2.4 RNA sequencing

The expression levels and types of IC (HAVCR2, CD244, PTGER4, LAG3, PDCD1, CD274, CD101, CTLA-4, CD160, LILRB4, and TIGIT) were analyzed in formalin-fixed and paraffin-embedded samples from 20 patients with *de novo* AITL using RNA sequencing. RNA sequencing, quality control, and data normalization were performed by Shanghai Rightongene Biotechnology Co., Ltd. (Shanghai, China). RNA sequencing data for AITL (n = 51) (GSE19069 and GSE58445) were downloaded from the GEO website (https://www.ncbi.nlm.nih.gov/gds/). The details of RNA sequencing are provided in the **Supplementary Materials**.

### 2.5 Analysis of exhaustion-related biological changes in CD8^+^TILs by RNA sequencing

The average expression levels of CD8A, CD8B, GZMA, GZMB, and PRF1 detected by RNA sequencing were defined as the cytotoxic T lymphocyte (CTL) levels (8); the 20 AITL cases were divided into high- and low-CTL groups based on the mean CTL marker levels. The 189-exhaustion biology-related gene panel was established according to a previous study, which showed a positive association with CD8^+^TILs exhaustion and reflected exhaustion from 19 biological aspects (9, 10) (including IC, glycosylation, effector functions, IFN response, translation, cytokines, ubiquitination, mitochondria, chromatin/DNA repair, proteases, homing and migration, cytoskeleton/cell adhesion, metabolism, cell surface receptors and ligands, apoptosis/cell death/caspase and annexins, membrane biology and vesicle transport, cell cycle, signaling, and miscellaneous aspects) (**Supplementary Table 1**). The 189-exhaustion biology-related gene panel was then used to analyze exhaustion-related biological changes in CD8^+^TILs from the high- and low-CTL groups.

### 2.6 Statistical analyses

Statistical analyses were performed using the Statistical Package for the Social Sciences (SPSS) software (version 26.0, SPSS Corp., Chicago, IL, USA). Spearman’s correlation coefficients were used to assess correlations among CD8^+^TILs, CD8A, CD8B, and IC. The chi-square test was used to compare the proportion of increased IC between the high- and low-CD8^+^TILs groups, as well as the CTL groups, and the differentially expressed genes (DEGs) between the high- and low-CTL groups. The cutoff values for CD8^+^TILs and CTL were defined as the points at which sensitivity and specificity were maximized in the receiver operating characteristic (ROC) curves for predicting overall survival (OS). Survival time was calculated using Kaplan-Meier analysis. A two-tailed *p* value less than 0.05 was considered statistically significant.

## 3 Results

### 3.1 Patient characteristics

Based on the inclusion criteria, 40 patients with *de novo* AITL were included in our cohort. The median patient age was 60 years (range, 38–83 years). Men accounted for 65% (n = 26) and women accounted for 35% (n = 14) of the sample. Eighteen (64.3%) patients were at an advanced stage (III/IV), and 28 (75.7%) were in a generally good condition (PS 0-1). The basic information is summarized in **Table 2**. Most patients (n = 24, 64.9%) received chemotherapy with the CHOP, R-CHOP, or ICE regimens, whereas a few patients (n = 5, 13.5%) received other treatment options, including surgery and immune-related therapies. Overall, 19 (73.1%) patients responded positively to the treatment. Eight patients (21.6%) refused treatment after diagnosis because of advanced disease stage, compromised general condition, and chemotherapy intolerance. Treatment-related data for three patients were not available.

**Table 2:** The baseline of patients with AITL in FCM and RNA sequencing.

Characteristic	NO	in group (%)
Age, median (range)	60(38-83)	
Gender		
Male	26/40	65
Female	14/40	35
PS		
0-1	28/37	75.7
2-5	9/37	24.3
Stage		
I/II	10/28	35.7
III/IV	18/28	64.3
IPI		
0-1	10/28	35.7
2-5	18/28	64.3
B-symptom		
yes	23/38	60.5
no	15/38	39.5
Extranodal involvement		
none	32/36	88.9
>=1	4/36	11.1
LDH>220 IU/L		
yes	17/28	60.7
no	11/28	39.3
Treatment		
Chemotherapy	24/37	64.9
Other therapy	5/37	13.5
No treatment	8/37	21.6
Response to treatment (CR+PR)	19/26	73.1
No response to treatment (SD+PD)	7/26	26.9

CR, complete remission; PR, partial remission; SD, stable disease; PD, progressive disease.

### 3.2 Analysis of IC in CD8^+^TILs using scRNA-seq

**Figure 1:**
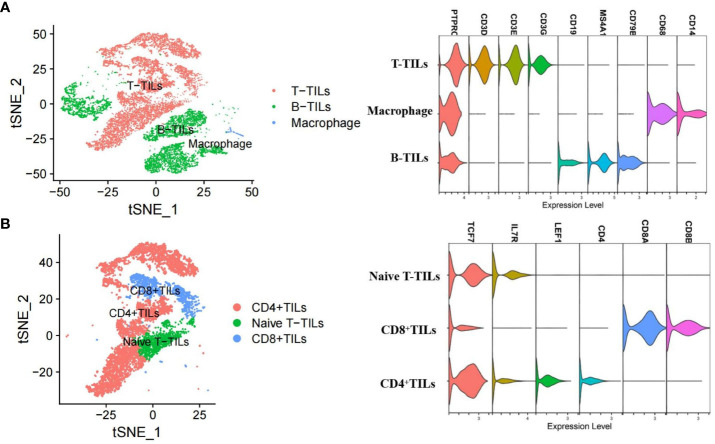
T-SNE analysis and marker genes for three major cell types **(A)** and three T-TILs types **(B)** in AITL samples detected using scRNA-seq (n = 2). The major cell types included T-TILs (CD3D, CD3E, CD3G), B-TILs (CD19, MS4A1, CD79B) and macrophages (CD68, CD14). The T-TILs comprised naive T-TILs (IL7R, TCF7), CD4^+^TILs (CD4) and CD8^+^TILs (CD8A, CD8B).

**Figure 2:**
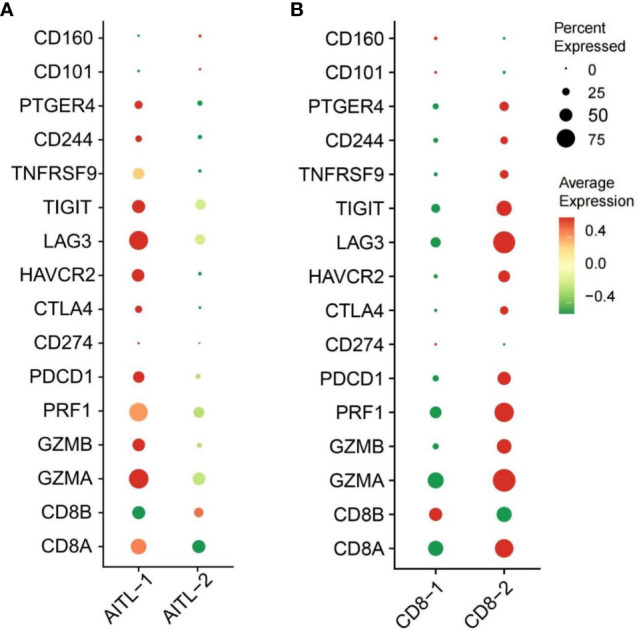
**(A)** The expression of CTL and IC genes in CD8^+^TILs from AITL-1 and AITL-2 detected using scRNA-seq, **(B)** The expression of CTL and IC genes in CD8-1 cells and CD8-2 cells detected using scRNA-seq (n = 2). The CTL genes included CD8A, CD8B, GZMA, GZMB, and PRF1. The IC genes included HAVCR2, CD244, PTGER4, LAG3, PDCD1, CD274, CD101, CTLA-4, CD160, LILRB4, and TIGIT.

After quality control and filtering, we obtained single-cell transcriptomes for 14 406 cells from the lymph node tissues of two patients with AITL (AITL-1, CD8^+^TILs:60.9%; AITL-2, CD8^+^TILs:48.6%). Through principal component analysis (PCA), t-distributed stochastic neighborhood embedding analysis (t-SNE), and uniform manifold approximation and projection (UMAP), three main clusters were identified, including T-TILs (CD3D, CD3E, CD3G), B-TILs (CD19, MS4A1, CD79B), and macrophages (CD68, CD14), among which the T-TILs comprised naive T-TILs (IL7R, TCF7), CD4^+^TILs (CD4), and CD8^+^TILs (CD8A, CD8B) (**Figure 1**; **Supplementary Figures 1**, **2**). AITL-1 showed higher expression of CTL and multiple IC genes in CD8^+^TILs than those in AITL-2 (**Figure 2A**); CD8^+^TILs from AITL-1 and AITL-2 were further divided into two clusters, including CD8-1 and CD8-2 cells (**Supplementary Figure 3**). Compared to that in CD8-1 cells, the expression of CTL and multiple IC genes was higher in CD8-2 cells (**Figure 2B**).

### 3.3 Analysis of IC in CD8^+^TILs using FCM

**Figure 3:**
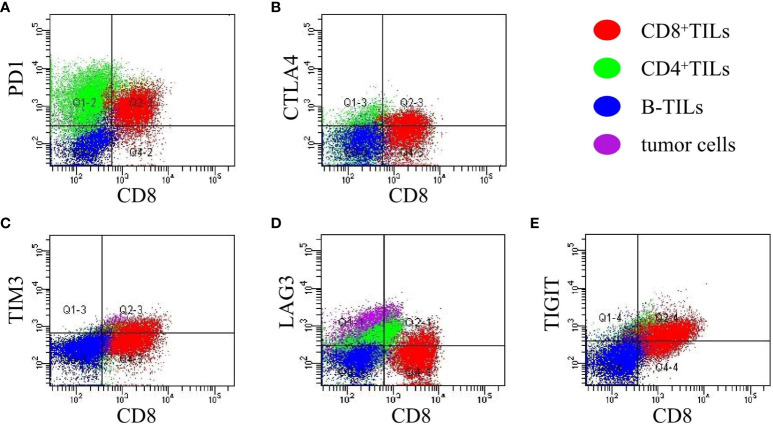
The expression of CD8^+^PD-1^+^, CD8^+^CTLA-4^+^, CD8^+^TIM3^+^, CD8^+^LAG3^+^ and CD8^+^TIGIT^+^ in AITL **(A–E)** detected using FCM (n = 20). The expression of IC was analyzed in CD8^+^TILs identified and gated by the CD3/CD8 markers. Red: CD8^+^TILs, Green: CD4^+^TILs, Blue: B-TILs, Purple: tumor cells.

**Figure 4:**
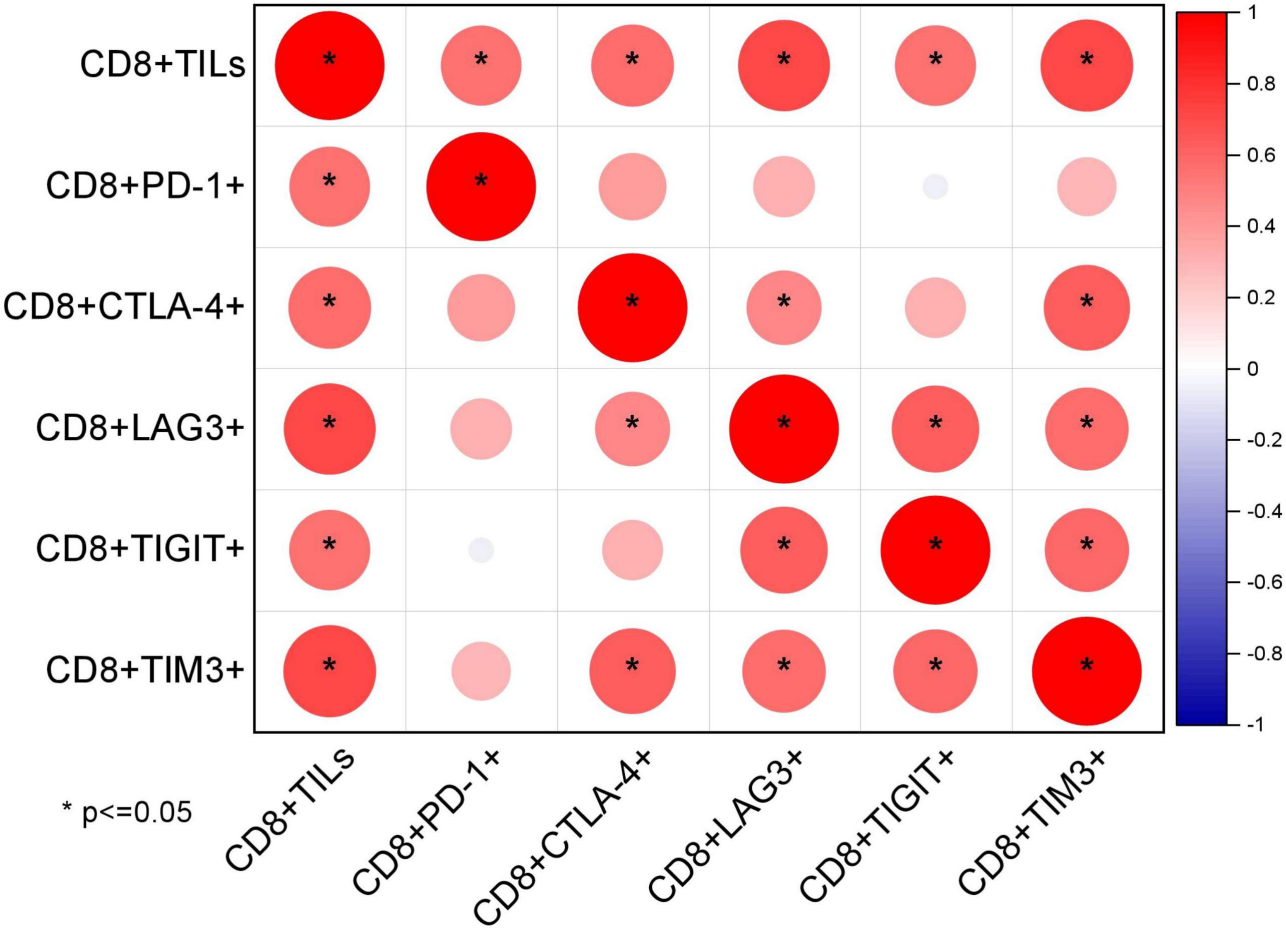
Correlations between the proportion of CD8^+^TILs and IC detected using FCM (n = 20) through Spearman’s test.

The mean proportions of CD8^+^TILs, CD8^+^PD-1^+^, CD8^+^CTLA-4^+^, CD8^+^TIM3^+^, CD8^+^LAG3^+^, and CD8^+^TIGIT^+^ in AITL (n = 20) (**Figures 3A–E**) were 40.75% (range, 4.1–78.9%), 14.39% (range, 0.19–45.53%), 9.09% (range, 0.11–44.28%), 2.63% (range, 0–10.66%), 3.36% (range, 0.22–14.93%), and 13.57% (range, 0.24–47.88%), respectively (**Table 3**). The proportion of CD8^+^TILs in AITL was positively correlated with that of CD8^+^TIM3^+^ (r = 0.72, *p* < 0.001), CD8^+^LAG3^+^ (r = 0.70, *p* < 0.001), CD8^+^CTLA-4^+^ (r = 0.57, *p* = 0.008), CD8^+^TIGIT^+^ (r = 0.55, *p* = 0.012), and CD8^+^PD-1^+^ (r = 0.54, *p* = 0.03) (**Figure 4**). Twenty patients were divided into high- and low-expression groups according to the mean proportions of CD8^+^TILs and IC, respectively. Compared to the low CD8^+^TILs group, the number of cases with IC ≥ 3 increased in the high CD8^+^TILs group (20% *vs*. 90%, *p* = 0.003) (**Supplementary Table 2**).

**Table 3:** The expression of IC on CD8^+^TILs in AITL.

IC	Mean (range)
CD8^+^TILs/T-TILs	40.57(4.1–78.9)
CD8^+^PD-1^+^/CD8^+^TILs	14.39(0.19–45.53)
CD8^+^CTLA-4^+^/CD8^+^TILs	9.09(0.11–44.28)
CD8^+^TIM3^+^/CD8^+^TILs	2.63(0–10.66)
CD8^+^LAG3^+^/CD8^+^TILs	3.36(0.22–14.93)
CD8^+^TIGIT^+^/CD8^+^TILs	13.57(0.24–47.88)

### 3.4 Analysis of IC in CD8^+^TILs using RNA sequencing

**Figure 5:**
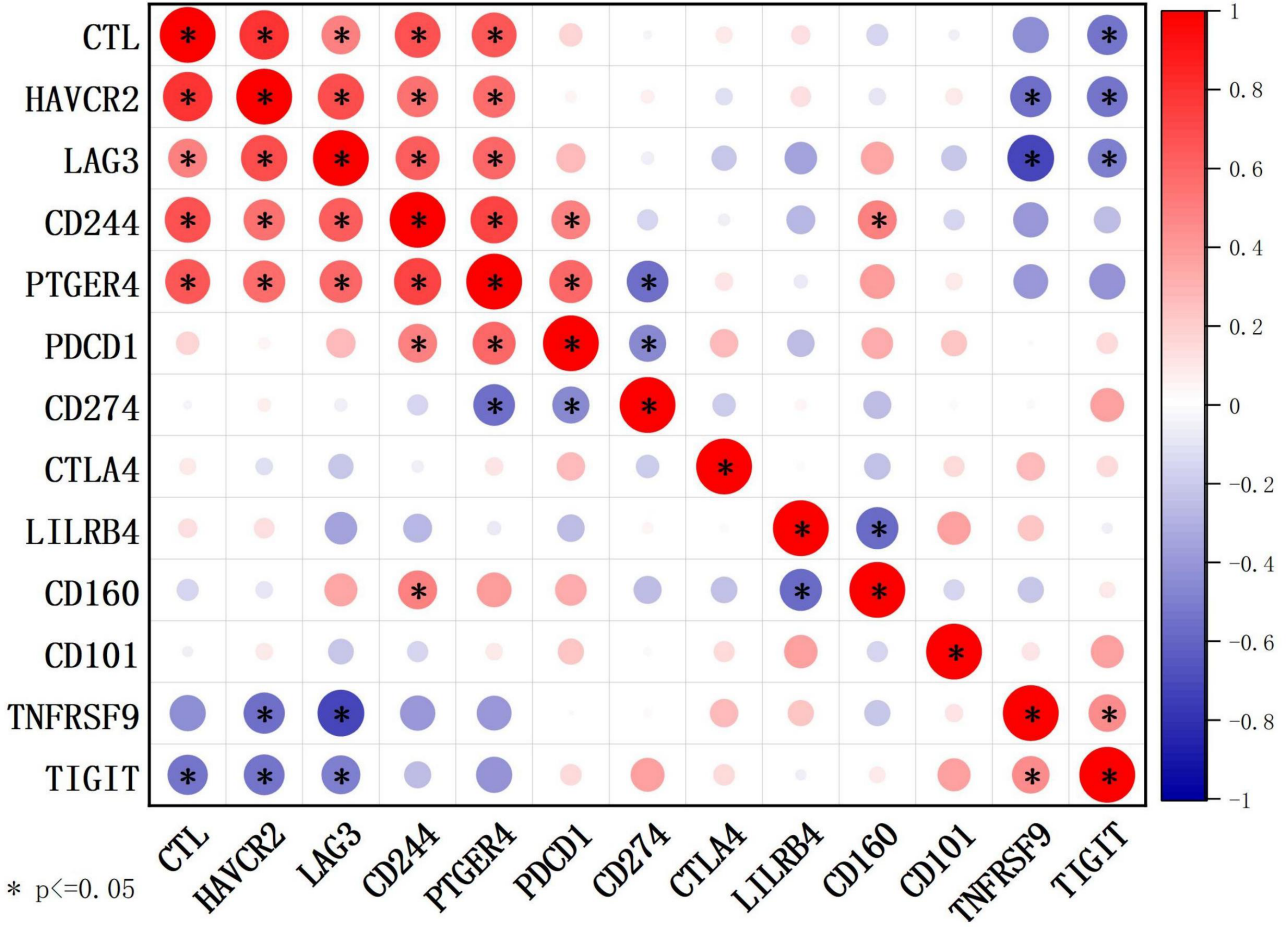
Correlations between CTL levels and IC genes detected using RNA sequencing (n = 20) through Spearman’s test. The CTL level was defined as the average expression of CD8A, CD8B, GZMA, GZMB, and PRF1 detected by RNA sequencing.

The mean expression levels of the CTL and IC genes are shown in **Supplementary Table 3**. The CTL showed strong positive correlations with HAVCR2, LAG3, PTGER4, and CD244 (**Figure 5**). Further, 20 patients were divided into high- and low-expression groups according to the mean expression of CTL and IC, respectively. Compared to those in the low CTL group, the number of cases with IC ≥ 6 tended to increase in the high CTL group (8.3% *vs*. 50%, *p* = 0.058) (**Supplementary Table 3**).

### 3.5 Analysis of exhaustion-related biological changes in CD8^+^TILs using RNA sequencing

**Figure 6:**
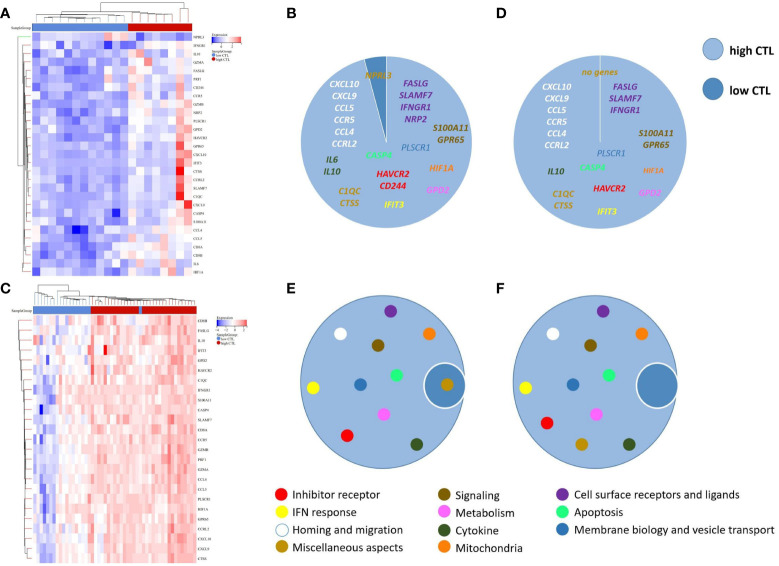
**(A)** Unsupervised hierarchical clustering of the 20 cases of AITL in our research, **(B)** The composition of DEGs between the high-CTL group (n = 8) and low-CTL group (n = 12) in our research, **(C)** Unsupervised hierarchical clustering of the 51 cases in the study by Iqbal et al. (GSE19069 and GSE58445), **(D)** The composition of DEGs between the high-CTL group (n=32) and low-CTL group (n = 19) in the study by Iqbal et al., **(E)** The composition of the biological aspects of DEGs in the high- and low-CTL groups in our research, **(F)** The composition of the biological aspects of DEGs in the high- and low-CTL groups in the study by Iqbal et al. The 189-exhaustion biology-related gene panel used in this study was established according to previous research and was applied to analyze the exhaustion-related biological changes in CD8^+^TILs from the high- and low-CTL groups.

**Figure 7:**
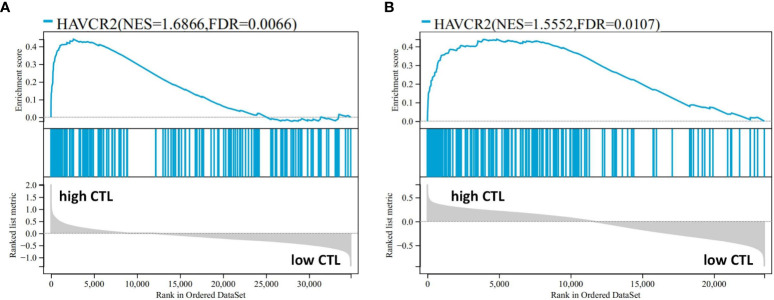
The result of GSEA performed in the high- and low-CTL groups after the 189-exhaustion biology-related gene panel in our data **(A)** and the study by Iqbal et al. (GSE19069 and GSE58445) **(B)**.

The mean expression levels of CTL-related genes (CD8A, CD8B, GZMA, GZMB, and PRF1) (8) was 27.84 (range, 10.59–65.13). The 20 cases were divided into a high-CTL group (n = 8) and low CTL group (n = 12) based on the mean CTL level. Unsupervised hierarchical clustering revealed two distinct clusters of exhaustive biology-related genes, consistent with the two different CTL-level groups (**Figure 6A**). Twenty-four DEGs were distributed across 11 biological aspects between the high- and low-CTL groups. Further analysis of the DEG characteristics showed that among the total DEGs, 23 (95.8%) DEGs distributed in 11 aspects were involved in the high CTL group, including IC (HAVCR2, CD244), signaling (S100A11, GPR65), mitochondria (HIF1A), cytokines (IL-6 and IL-10), cell surface receptors and ligands (FASLG, SLAMF7, IFNGR1, NRP2), homing and migration (CXCL10, CXCL9, CCL5, CCR5, CCL4, CCRL2), membrane biology and vesicle transport (PLSCR1), apoptosis/cell death/caspase and annexin (CASP4), metabolism (GPD2), IFN response (IFIT3), and miscellaneous aspects (C1QC, CTSS). One (4.2%) DEG (NPRL3), distributed in miscellaneous aspects, was detected in the low-CTL group. Chi-square analysis revealed that more DEGs were involved in the high CTL group (*p* = 0) and covered more biological aspects than the low CTL group (*p* = 0.004) (**Figures 6B, E** and **Table 4**). Besides, the result of gene-set enrichment analysis (GSEA) demonstrated that exhaustion biology-related genes were enriched in the high CTL group from our dataset (FDR = 0.005) (**Figure 7A**).

**Table 4:** The composition of DEGs in our dataset and in the datasets from the study by Iqbal et al. (GSE19069 and GSE58445).

Dataset	DEGs	high CTL	Biological aspects	low CTL	Biological aspects	*p^1^*	*p^2^*
Our dataset	24	23(95.8.%)	11	1(4.2%)	1	0	0.004
Iqbal’s dataset	20	20(100%)	11	0(0%)	0	0	0

DEGs, differentially expressed genes.

*p^1^*: DEGs in high CTL vs low CTL, *p^2^*: Biological aspects of DEGs in high CTL vs low CTL.

Following the protocol described above, AITL cases (n = 51) from the data by Iqbal et al. (GSE19069 and GSE58445) were divided into a high-CTL group (32 cases) and low-CTL group (19 cases) based on the mean CTL level (10.61). The pivotal 24 DEGs obtained from our results were compared between the two groups. Clustering results revealed two distinct clusters (**Figure 6C**). All 20 DEGs (distributed across 11 biological aspects) were involved in the high CTL group, including IC (HAVCR2), signaling (S100A11, GPR65), mitochondria (HIF1A), cytokines (IL-10), cell surface receptors and ligands (FASLG, SLAMF7, IFNGR1), homing and migration (CXCL10, CXCL9, CCL5, CCR5, CCL4, CCRL2), membrane biology and vesicle transport (PLSCR1), apoptosis/cell death/caspase and annexin (CASP4), metabolism (GPD2), IFN response (IFIT3), and miscellaneous aspects (C1QC, CTSS). The chi-square analysis revealed that more DEGs were involved in the high-CTL group (*p* = 0) and covered more biological aspects than those in the low-CTL group (*p*=0) (**Figures 6D, F** and **Table 4**). One case in the low-CTL group clustered with the high-CTL group in the study by Iqbal et al. (**Figure 6C**). Further, the results of GSEA demonstrated that exhaustion biology-related genes were enriched in the high-CTL group from the dataset of Iqbal et al. (FDR = 0.006) (**Figure 7B**).

### 3.6 CD8^+^TILs, CTL and correlation with clinical features

The correlations between the proportion of CD8^+^TILs, CTL levels, and clinical features are shown in **Table 5**. Patients in the high CD8^+^TILs and high CTL groups were prone to demonstrate a lower response rate (CR + PR) to treatment and presented with more stage IV disease compared with those in the low CD8^+^TILs and low CTL groups.

**Table 5:** Patient characteristics according to CD8^+^TILs and CTL levels.

Characteristic	high CD8^+^TILs (n=6)	low CD8^+^TILs (n=14)	*p*	high CTL (n=8)	low CTL (n=12)	*p*
Age, median (range)	64.5(38-83)	58(45-75)		62(53-75)	56(45-69)	
Age			0.314			0.65
>=60 years	4/6(66.7%)	6/14(42.9%)		5/8(62.5%)	5/12(41.6%)	
<60 years	2/6(33.3%)	8/14(57.1%)		3/8((37.5%)	7/12(58.4%)	
Gender			0.187			0.325
Male	5/6(83.3%)	7/14(50%)		7/8(87.5%)	7/12(58.4%)	
Female	1/6(16.7%)	7/14(50%)		1/8(12.5%)	5/12(41.6%)	
PS			0.237			0.608
0-1	3/5(60%)	4/14(28.6%)		5/8(62.5%)	8/10(80%)	
2-5	2/5(40%)	10/14(71.4%)		3/8(37.5%)	2/10(20%)	
Stage			0.09			0.141
I/II/III	2/6(33.3%)	10/13(76.9%)		4/8(50%)	7/8(87.5%)	
IV	4/6(66.7%)	3/13(23.1%)		4/8(50%)	1/8(12.5%)	
IPI			0.636			1
0-1	1/5(20%)	2/7(28.6%)		3/8(37.5%)	4/9(44.4%)	
2-5	4/5(80%)	5/7(71.4%)		5/8(62.5%)	5/9(55.6%)	
B-symptom			0.455			0.367
yes	3/6(50%)	9/14(64.3%)		6/8(75%)	5/10(50%)	
no	3/6(50%)	5/14(35.7%)		2/8((25%)	5/10(50%)	
Extranodal involvement			0.479			0.385
none	6/6(100%)	12/14(85.7%)		4/6(66.7%)	7/8(87.5%)	
>=1	0/6(0%)	2/14(14.3%)		2/6(33.3%)	1/8(12.5%)	
LDH>220 IU/L			0.566			0.565
yes	3/5(60%)	7/10(70%)		4/5(80%)	4/8(50%)	
no	2/5(40%)	3/10(30%)		1/5(20%)	4/8(50%)	
Response to treatment			0.093			0.07
CR+PR	1/3(33.3%)	10/11(90.9%)		3/6(50%)	7/7(100%)	
SD+PD	2/3(66.7%)	1/11(9.1%)		3/6(50%)	0/7(0%)	

### 3.7 Survival and prognostic analysis

**Figure 8:**
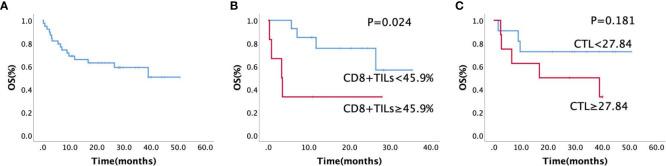
**(A)** Kaplan–Meier estimates of overall survival rate for all the patients (n = 40), **(B)** Kaplan–Meier estimates of the overall survival rate for CD8^+^TILs by FCM (n = 20, *p* = 0.024), **(C)** Kaplan–Meier estimates of the overall survival rate for different CTL levels by RNA sequencing (n = 20, *p =* 0.181).

Overall, follow-up data was available for 39 patients, with a follow-up rate of 97.5% (39/40). The follow-up time ranged from 0.3 to 38.5 months, and the median OS time was 24.7 months (95% confidence interval: 19.39–30.01 months; **Figure 8A**). High CD8^+^TILs (≥ 45.9%) detected by FCM (*p* = 0.024, **Figure 8B**) was a significant risk factor for patients with AITL; patients with high CTL levels (≥ 27.84) detected by RNA-seq (*p* = 0.181, **Figure 8C**) also tended to have a poor prognosis.

## 4 Discussion

AITL has a complex tumor microenvironment (TME), in which tumor cells share close crosstalk with other immune cells, particularly TILs. Among TILs, CD8^+^TILs are the primary components that deliver anti-tumor responses (11, 12) and serve as favorable prognosticators in many tumors (13). CD8^+^TILs can exert anti-tumor functions (11) through several pathways such as the release of GZMA and GZMB, Fas-Fas ligand pathway-mediated apoptosis (14, 15), and secretion of inflammatory cytokines like IFN-γ (16) and TNF-α (17). We previously found that an increase in CD8^+^TILs had a negative impact on the prognosis of AITL at the protein level (3, 4), and hypothesized that CD8^+^TILs are exhausted in AITL. Intriguingly, Pritchett et al. recently suggested the presence of exhausted CD8^+^TILs in AITL, characterized by IC overexpression (18) compared with those in healthy controls. However, the exhaustion status of CD8^+^TILs among patients with AITL and their clinical significance remain unknown. In this study, we comprehensively investigated the exhaustion status of CD8^+^TILs based on the expression of IC and multilevel analysis of exhaustion biology-related genes, as well as their impact on prognosis, to better understand the exhaustion status of CD8^+^TILs in AITL, which is beneficial for developing targeted immunotherapy.

IC overexpression is a hallmark of CD8^+^TILs exhaustion (19). By interacting with the corresponding ligands expressed on tumor cells and/or antigen-presenting cells, IC can activate signaling-related pathways and interfere with the metabolism and mitochondrial function of T lymphocytes. These changes eventually induce T lymphocytes exhaustion and impair effector function, resulting in tumor progression (20). Our scRNA-seq study found that there were two clusters of CD8^+^TILs from AITL, and the expression of multiple IC genes in one cluster (CD8-2 cells) was markedly increased compared with that in the other cluster (CD8-1 cells). Meanwhile, the proportion of CD8-2 cells was higher in AITL cases with more CD8^+^TILs, suggesting that the proportions of both CD8^+^TILs and exhausted CD8^+^TILs was increased in AITL. Further analysis demonstrated that the proportion of CD8^+^TILs positively correlated with the expression as well as types of IC at the protein and transcription levels, indicating that the more CD8^+^TILs, the more exhausted CD8^+^TILs were induced by the overexpression of IC (21) in AITL. This could impair anti-tumor function and be the main cause of poor prognosis in patients with high CD8^+^TILs and CTL levels, which has not been reported so far.

To further explore the relationship between the exhaustion status of CD8^+^TILs and their possible negative impact on prognosis in AITL, we evaluated the functional status associated with CD8^+^TILs exhaustion from multiple biological aspects (9, 10) at the gene transcription level to reveal the anti-tumor function of CD8^+^TILs within the high- and low-CTL groups. Our data as well the data from the study of 51 AITL cases by Iqbal et al. (GSE19069 and GSE58445), the largest sample size of RNA sequencing for AITL to date, revealed that CD8^+^TILs in the high CTL group were more prone to exhaustion-related biological changes and were involved in regulating exhaustion, the obvious deficiency in biological function, and immune suppression of the TME. On the one hand, inhibitory cytokines, cell surface receptors, and abnormal signal transduction-related changes may participate in regulating CD8^+^TILs exhaustion, such as by inducing the expression of exhaustion-related transcription factors (22–25), and inhibiting the activation and cytokine secretion of CD8^+^TILs (20, 26). On the other hand, there were obvious deficiencies in the biological function of CD8^+^TILs in the high CTL group, characterized by the dysregulated expression of genes related to metabolism, mitochondria, and effector function in CD8^+^TILs, suggesting a disturbance in normal metabolic processes (27), mitochondrial dysfunction, and deficiency in energetic adaptations (28, 29). Further, several chemokines were enriched in the high-CTL group, which could recruit M2 macrophages (30) and Tregs (31), thereby enhancing immune suppression in the TME (4) and aggravating the exhaustion of CD8^+^TILs. In summary, exhausted CD8^+^TILs in the high CTL group exhibited abnormal biological changes in addition to high IC expression, suggesting a more severe degree of CD8^+^TILs exhaustion in the high CTL group than in the low CTL group, eventually resulting in tumor proliferation (32) and poor prognosis. Notably, one case in the low CTL group showed severe exhaustion of CD8^+^TILs, indicating the need for a detailed investigation.

In addition to predicting survival risk, our study is helpful for dynamically monitoring the anti-tumor function of CD8^+^TILs in patients based on their exhaustion status in AITL and can provide suggestions for developing individualized therapeutic approaches. Patients with AITL showing more exhausted CD8^+^TILs should be screened for poor prognosis, especially in case of high CD8^+^TILs and CTL levels, and the most important and ideal treatment is to recover the effector function of CD8^+^TILs. Immunotherapy targeting IC (33) and immunosuppressive cytokines (34) can also be adopted in combination with routine chemotherapy to improve patient prognosis. Furthermore, a high proportion of CD8^+^TILs and CTL levels can also serve as novel indicators of treatment response.

Interestingly, we identified a few cases with high CD8^+^TILs or high CTL levels who showed good prognosis (data not shown), and CD8^+^TILs from these cases demonstrated low expression and few types of IC. These results imply that the high infiltration of normal CD8^+^TILs in AITL can effectively exert anti-tumor function, which is valuable in designing individualized immunotherapy for patients with AITL and needs further exploration.

**Figure 9:**
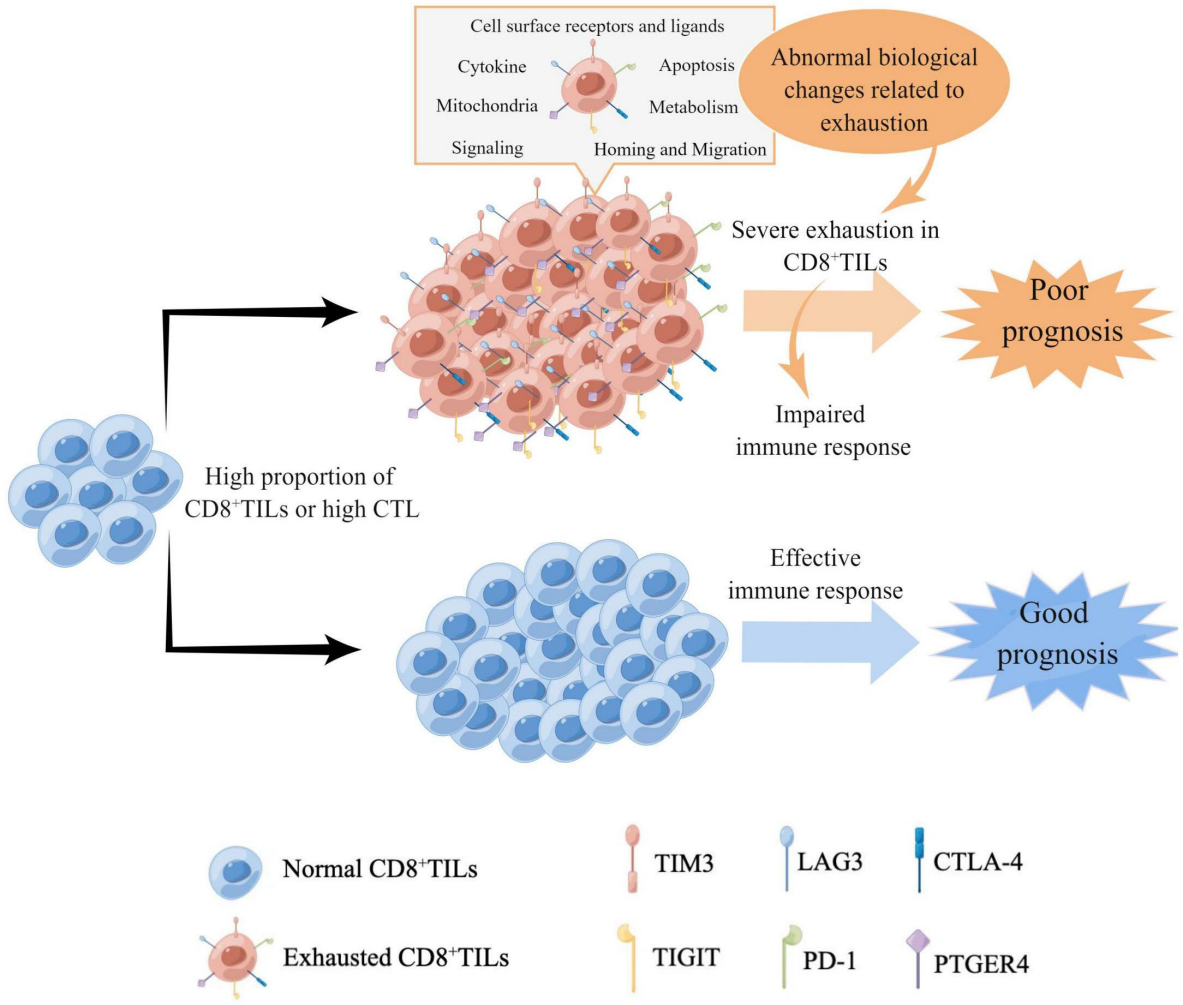
The model of the characteristics of CD8^+^TILs exhaustion status in AITL. High proportions of CD8^+^TILs or high CTL in AITL indicated higher expression level and more types of IC, accompanied by extensive biological changes related to exhaustion, which suggested severe exhaustion of CD8^+^TILs and impairment of immune responses, resulting in poor prognosis. Meanwhile, the high proportion of CD8^+^TILs or high CTL with low expression of IC indicated good prognosis in AITL, suggesting that the normal CD8^+^TILs could effectively exert anti-tumor functions.

In conclusion, our research demonstrated that the exhaustion status of CD8^+^TILs in AITL was characterized by the high expression of multiple IC. The greater the number of CD8^+^TILs, the greater was the proportion of severely exhausted CD8^+^TILs, accompanied by extensive exhaustion-related biological changes, which may be one of the main reasons underlying the poor prognosis of patients with high CD8^+^TILs and CTL (**Figure 9**). Our findings regarding CD8^+^TILs exhaustion status could not only help predict the survival risk of patients with AITL but also facilitate further mechanistic studies and are valuable in guiding immunotherapy strategies.

## Data availability statement

The datasets presented in this study can be found in online repositories. The names of the repository/repositories and accession number(s) can be found below: China National Center for Bioinformation, https://ngdc.cncb.ac.cn/omix/preview/dgy192YH, accession number: OMIX004884.

## Ethics statement

The studies involving humans were approved by The Ethics Committee on Biomedical Research, West China Hospital of Sichuan University. The studies were conducted in accordance with the local legislation and institutional requirements. The ethics committee/institutional review board waived the requirement of written informed consent for participation from the participants or the participants’ legal guardians/next of kin. The data for the patients included in the study were retrospectively analyzed.

## Author contributions

SZ designed the study. QZ, XD, and ZC collected the data of flow cytometry. QZ and YMY analyzed and interpreted the data of flow cytometry and RNA sequencing. QZ and NC analyzed and interpreted the data of single-cell RNA sequencing. SZ, WL, YXY, WZ, and ZC contributed to clinical data. QZ and YMY prepared the figures and tables. SZ and QZ wrote the paper. All authors contributed to the article and approved the submitted version.

## Supplementary material

Supplementary Figure 1 UMAP and t-SNE analysis according to different major cell types (n = 3) and different patients with AITL (n = 2).

Supplementary Figure 2 UMAP and t-SNE analysis according to different T-TILs types (n = 3) and different patients with AITL (n = 2).

Supplementary Figure 3 UMAP and t-SNE analysis according to different CD8^+^TILs types (n = 2) and different patients with AITL (n = 2).
