# Supplementary figures and images for: High CD8+tumor-infiltrating lymphocytes indicate severe exhaustion and poor prognosis in angioimmunoblastic T-cell lymphoma
Source: Front Immunol. 2023 Sep 15;14:1228004. doi: 10.3389/fimmu.2023.1228004 (PMC10540231; doi:10.3389/fimmu.2023.1228004)

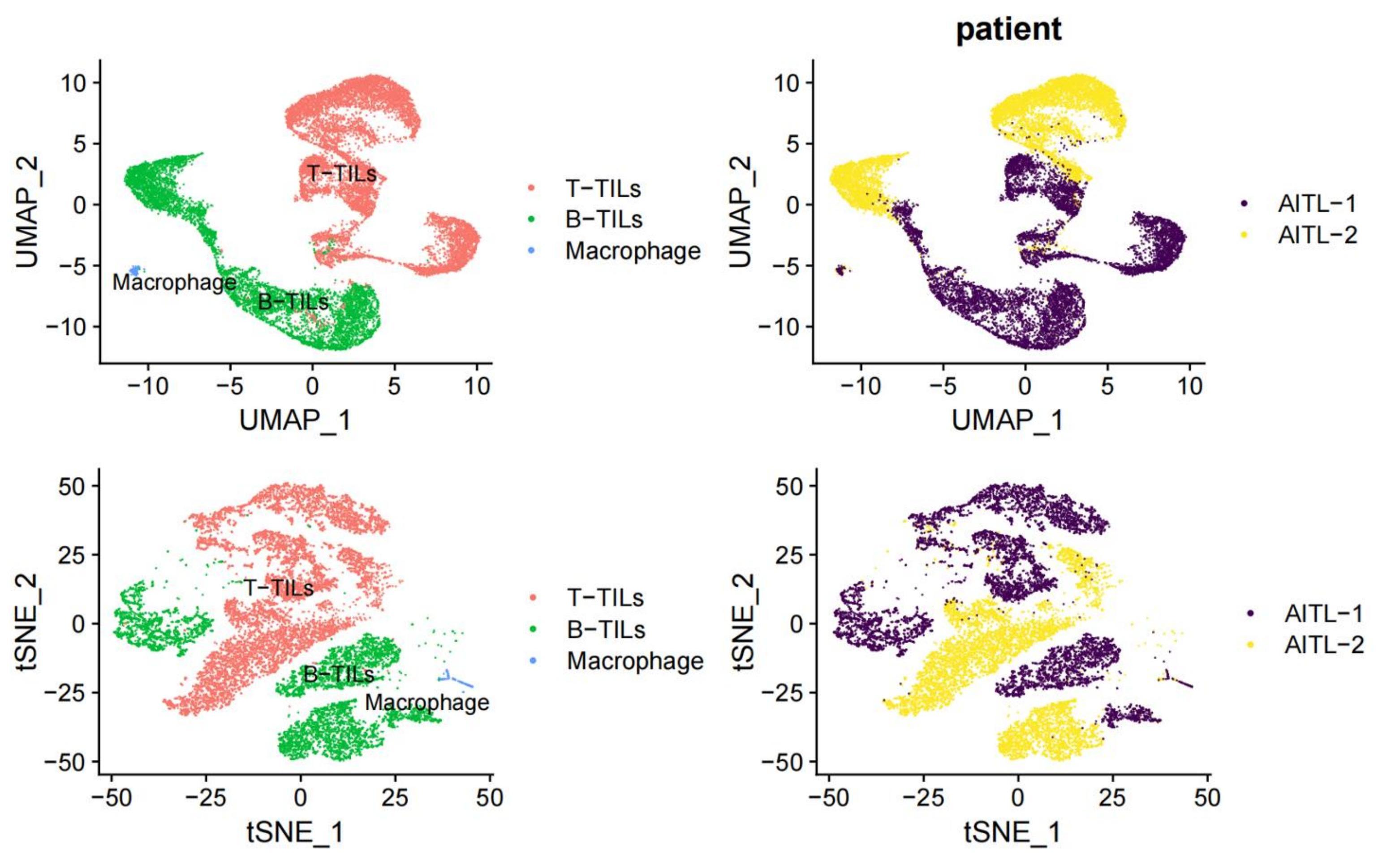

Supplement: Supplementary Figure 1 — UMAP and t-SNE analysis according to different major cell types (n = 3) and different patients with AITL (n = 2). [file Image_1.jpeg]

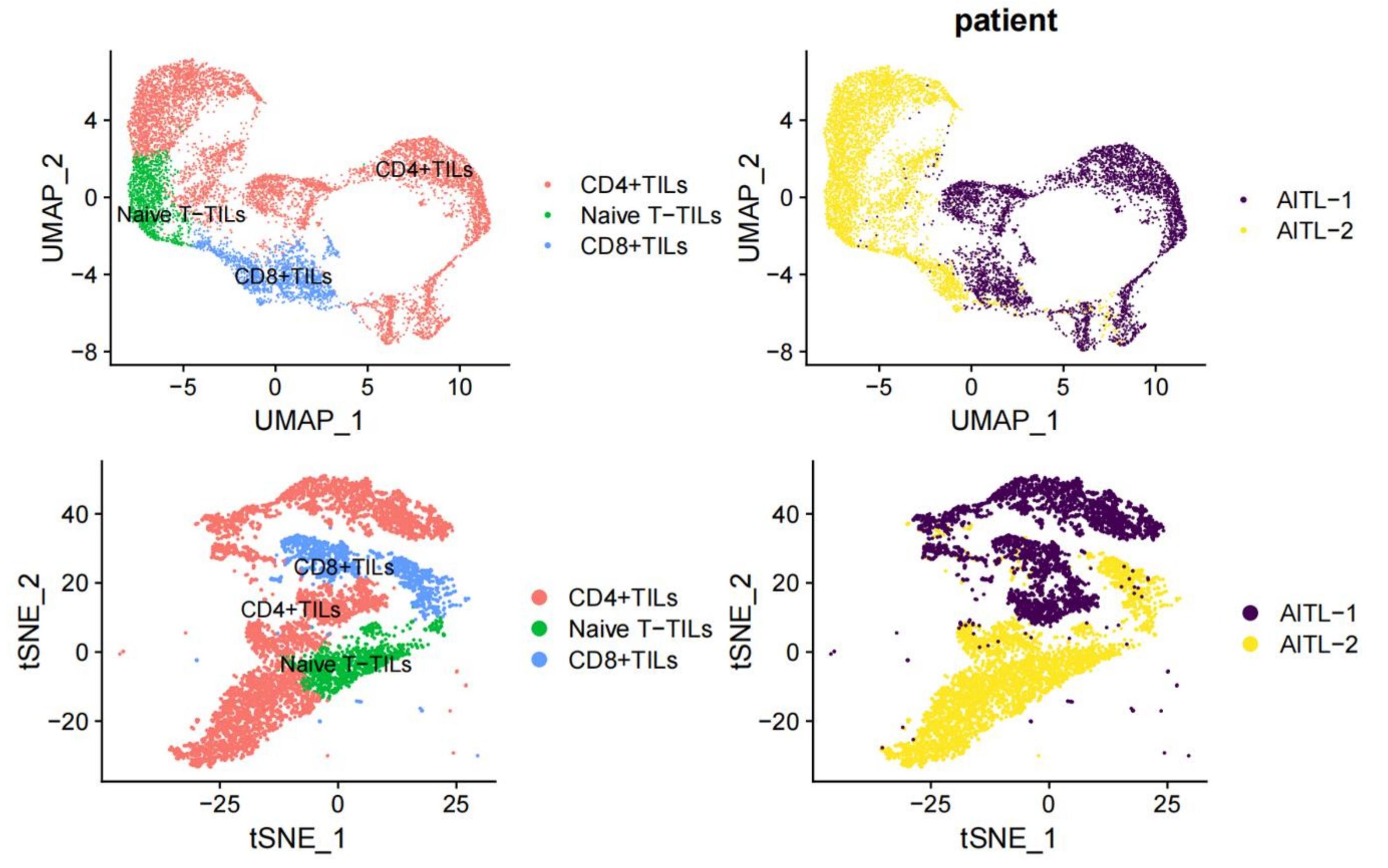

Supplement: Supplementary Figure 2 — UMAP and t-SNE analysis according to different T-TILs types (n = 3) and different patients with AITL (n = 2). [file Image_2.jpeg]

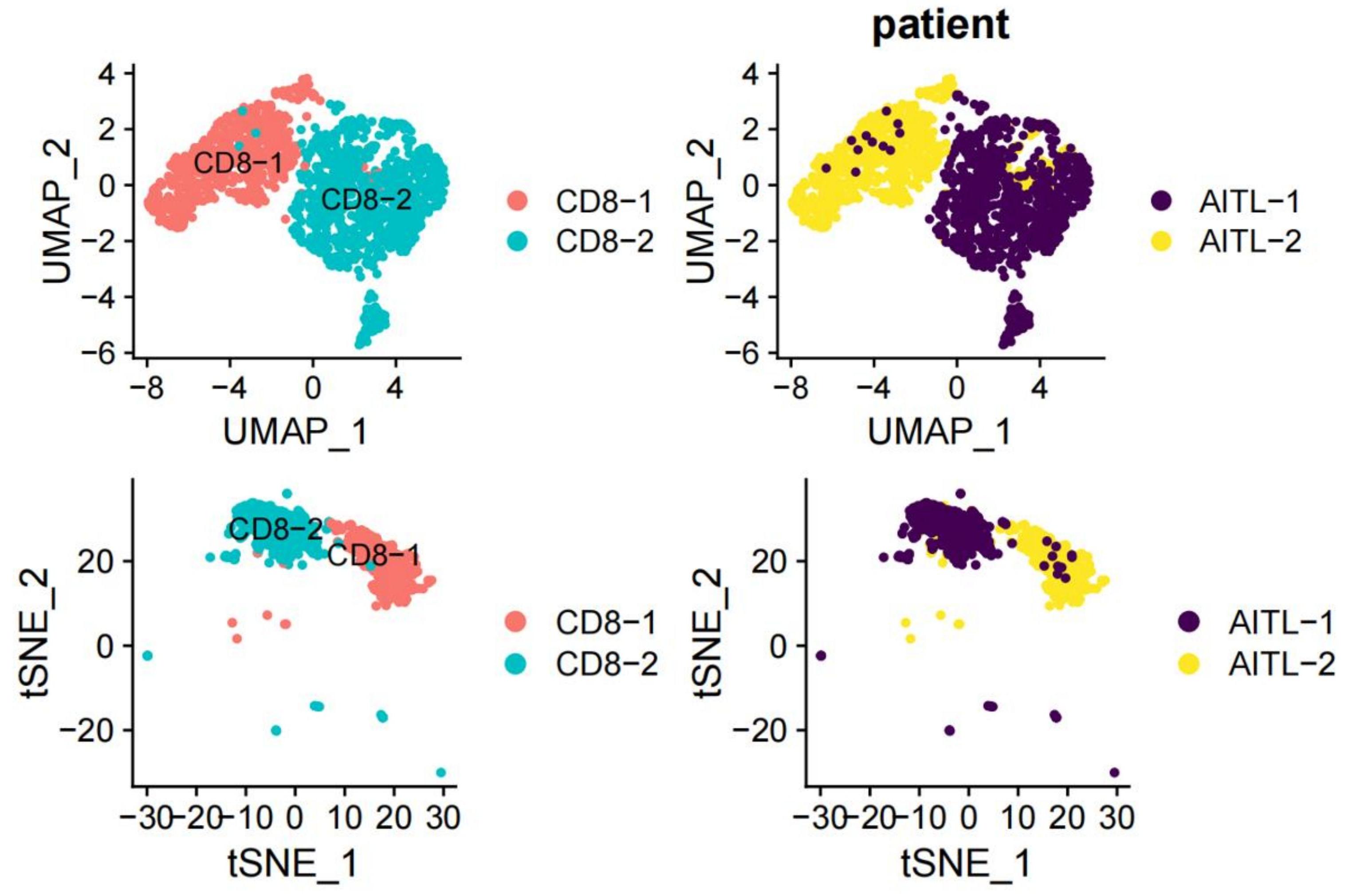

Supplement: Supplementary Figure 3 — UMAP and t-SNE analysis according to different CD8+TILs types (n = 2) and different patients with AITL (n = 2). [file Image_3.jpeg]
