# Supplementary material for: High CD8+tumor-infiltrating lymphocytes indicate severe exhaustion and poor prognosis in angioimmunoblastic T-cell lymphoma
Source: Front Immunol. 2023 Sep 15;14:1228004. doi: 10.3389/fimmu.2023.1228004 (PMC10540231; doi:10.3389/fimmu.2023.1228004)
Supplement: Supplementary file 4 [file Table_1.docx]

Supplementary Table 1. Molecular Signature of CD8^+^TILs exhaustion

| **Molecular Signature of CD8^+^TILs Exhaustion** | | | | |
| --- | --- | --- | --- | --- |
| **Immune checkpoint** | **Mitochondria** | **Homing and Migration** | **Cell surface receptors and ligands** | **Miscellaneous aspects** |
| CD244 | COCH | CXCR6 | TNFRSF9 | SAMD3 |
| PDCD1 | PPM1B | CCL3 | CD9 | TMEM50B |
| CD274 | TRIM47 | CCRL2 | ALCAM | F2R |
| LAG3 | NRIP1 | CCL4 | ITGAV | CTSS |
| CTLA4 | KLK6 | CCR5 | ITGA1 | DENND4A |
| LILRB4 | LY75 | CXCL10 | PGLYRP1 | GCH1 |
| PTGER4 | BUB1 | CXCR3 | ITM2A | TMEM88 |
| CD160 | NDFIP1 | CXCL9 | ADAM19 | PADI2 |
| TIGIT | SPRED2 | CCL5 | ITGB1 | ERN1 |
| HAVCR2 | CBX4 | TAP2 | VCAM1 | PHF20L1 |
| KLRG1 | EEA1 | **Cytoskeleton/Cell adhesion** | CD7 | C1QC |
| CD101 | FGL2 | ACTN1 | SLAMF6 | SPATA20 |
| **Signaling** | SPG21 | MACF1 | SLAMF7 | EIF1 |
| RGS16 | NUSAP1 | SPP1 | CD27 | NPRL3 |
| TANK | HIF1A | KRT77 | CYSLTR2 | FAM172A |
| PTPN13 | PON2 | AHNAK | ADRB2 | MAPRE3 |
| SH2D2A | TWSG1 | JAM2 | IFNGR1 | ADM2 |
| PLK4 | KIF23 | KRT80 | CD69 | CHD2 |
| GPR65 | PPIC | TPM3 | CLEC1B | RNF212 |
| FYN | FIGNL1 | NRP2 | FASLG | TMEM214 |
| JAK3 | **Chromatin/DNA Repair** | NRP1 | **Apoptosis, cell death, caspase and annexins** | ASB11 |
| S100A6 | TOP2A | LAYN | CASP3 | KCNA5 |
| S100A4 | RPA2 | **Metabolism** | PERP | BEST2 |
| S100A11 | ZFYVE27 | GPD2 | CASP4 | SH3BP4 |
| S100A13 | PHF21A | ENTPD1 | BCL2 | SLC29A4 |
| MAP3K4 | MUM1 | KPNA2 | CASP1 | ADIG |
| RGS1 | STAT4 | ART3 | **Membrane biology and vesicle transport** | MUC15 |
| CAMK2N1 | **Cell cycle** | ALDH2 | PLSCR1 | BIRC7 |
| PTPRE | MKI67 | SLC12A2 | **Glycosylation** | FUT4 |
| SFN | CKS2 | NDUFA5 | LGALS3 | CRYGS |
| RAB22A | GAS2 | P2RX4 | **Translation** | RIPPLY2 |
| PTGER1 | MAD2L1 | DNAJA4 | CPSF2 | **Effector functions** |
| GKAP1 | CHEK1 | CH25H | SFPQ | GZMA |
| KLK13 | PRC1 | ADSSL1 | **Proteases** | GZMK |
| PLEKHM3 | CCNB1 | APOE | SERPINB9 | GZMB |
| **Cytokines** | CCNB2 | DCT | RNF11 | PRF1 |
| IL4 | CDC6 | DHRS7C | **IFN Response** | CD8A |
| IL6 | CDC37 | PDE10A | ISG20 | CD8B |
| IL10 | **Ubiquitination** | FDFT1 | IRF4 | IFNG |
| TGFB1 | FBXO40 | GK2 | IFIT1 | TNF |
|  | OTUD7B |  | IFIT3 | CX3CR1 |
