# Supplementary material for: High CD8+tumor-infiltrating lymphocytes indicate severe exhaustion and poor prognosis in angioimmunoblastic T-cell lymphoma
Source: Front Immunol. 2023 Sep 15;14:1228004. doi: 10.3389/fimmu.2023.1228004 (PMC10540231; doi:10.3389/fimmu.2023.1228004)
Supplement: Supplementary file 5 [file Table_2.docx]

Supplementary Table 2. Correlation between the proportion of CD8^+^TILs and the type of IC analyzed using FCM

| cases | CD8^+^TILs  ≥40.75% | CD8^+^TIM3^+^  ≥2.63% | CD8^+^LAG3^+^  ≥3.36% | CD8^+^PD-1^+^  ≥14.39% | CD8^+^TIGIT^+^  ≥13.57% | CD8^+^CTLA-4^+^≥9.09% |
| --- | --- | --- | --- | --- | --- | --- |
| case 1 | + | + | + | - | + | + |
| case 2 | + | + | + | + | + | + |
| case 3 | + | + | + | + | + | + |
| case 4 | + | + | + | - | + | - |
| case 5 | + | + | + | + | + | - |
| case 6 | - | + | - | - | + | + |
| case 7 | - | - | + | - | + | - |
| case 8 | + | - | + | + | + | + |
| case 9 | - | - | - | - | + | - |
| case 10 | + | + | - | + | + | - |
| case 11 | + | - | + | - | - | - |
| case 12 | - | - | - | - | - | - |
| case 13 | + | + | - | + | - | + |
| case 14 | - | - | - | + | - | + |
| case 15 | - | + | + | + | - | + |
| case 16 | - | - | - | - | - | - |
| case 17 | + | + | + | + | - | + |
| case 18 | - | - | - | - | - | + |
| case 19 | - | - | - | + | - | - |
| case 20 | - | - | - | - | - | - |
