# Supplementary material for: High CD8+tumor-infiltrating lymphocytes indicate severe exhaustion and poor prognosis in angioimmunoblastic T-cell lymphoma
Source: Front Immunol. 2023 Sep 15;14:1228004. doi: 10.3389/fimmu.2023.1228004 (PMC10540231; doi:10.3389/fimmu.2023.1228004)
Supplement: Supplementary file 6 [file Table_3.docx]

Supplementary Table 3. Correlation between CTL levels and the type of IC analyzed using RNA sequencing

| cases | CTL  ≥27.84 | HAVCR  2≥10.61 | LAG3  ≥15.09 | PTGER4  ≥11.32 | CD244  ≥2.81 | PDCD1  ≥5.08 | CTLA-4  ≥24.15 | TIGIT  ≥14.29 | TNFRSF9  ≥10.54 | CD274  ≥32.31 | LILRB4  ≥2.55 | CD160  ≥1.86 | CD101  ≥2.63 |
| --- | --- | --- | --- | --- | --- | --- | --- | --- | --- | --- | --- | --- | --- |
| case 1 | - | - | - | - | - | - | - | - | - | + | + | - | + |
| case 2 | + | + | + | + | + | - | - | - | - | - | - | + | - |
| case 3 | - | - | - | - | - | - | - | - | + | - | + | - | + |
| case 4 | - | - | - | - | - | - | - | + | + | + | - | + | - |
| case 5 | - | - | - | - | - | - | + | + | + | - | - | + | + |
| case 6 | + | + | + | + | + | - | - | - | - | + | + | - | + |
| case 7 | - | - | - | - | - | - | - | + | + | + | + | - | - |
| case 8 | - | - | + | - | - | - | + | - | - | + | - | - | - |
| case 9 | + | + | - | - | - | - | + | - | + | - | + | - | - |
| case 10 | - | - | - | - | - | - | - | + | + | + | - | - | + |
| case 11 | + | + | + | - | - | - | - | - | - | + | - | - | - |
| case 12 | - | - | - | - | - | - | + | + | + | + | + | - | - |
| case 13 | + | + | + | + | + | - | + | - | - | + | - | - | - |
| case 14 | + | + | - | - | - | - | + | + | + | + | + | - | + |
| case 15 | + | + | + | + | - | - | - | - | - | - | - | - | - |
| case 16 | - | - | - | - | - | - | + | + | + | + | - | - | + |
| case 17 | - | - | - | + | + | + | + | - | + | - | - | - | - |
| case 18 | + | + | + | + | + | + | - | - | - | - | - | + | - |
| case 19 | - | - | - | - | - | - | - | - | + | - | + | - | - |
| case 20 | - | + | + | + | + | + | - | + | - | - | - | + | - |
