# Supplementary material for: High CD8+tumor-infiltrating lymphocytes indicate severe exhaustion and poor prognosis in angioimmunoblastic T-cell lymphoma
Source: Front Immunol. 2023 Sep 15;14:1228004. doi: 10.3389/fimmu.2023.1228004 (PMC10540231; doi:10.3389/fimmu.2023.1228004)
Supplement: Supplementary file 7 [file DataSheet_1.docx]

***Single-cell RNA sequencing (scRNA-seq)***

**Sample collection and processing**

scRNA-seq was performed in Department of Respiratory and Critical Care Medicine, Frontiers Science Center for Disease-related Molecular Network, West China Hospital, Sichuan University. Biopsy samples of two AITL with a medium size of 1cm×1cm×1cm were mechanically dissociated into single-cell suspensions and cryopreserved in RPMI 1640 medium with 10% DMSO in liquid nitrogen. Then the cells were were promptly thawed and washed for three times with phosphate-buffered saline (PBS), followed by lysis with 0.15 mol/L of ammonium chloride for 5 min. The viability of cells from each sample was confirmed to be over 85% and the final concentration was adjusted to ∼1200 cells/ul.

**Single-cell RNA library construction and sequencing**

Samples with appropriate volumes for a target capture of 8,000 cells were loaded into a microfluidic chip (Chromium Next GEM Single Cell 5' Reagent Kits v2 (Dual Index) 10x Genomics) and processed according to the manufacturer’s instruction. The cDNA was purified and cleaned up after the reverse transcription. Then the cDNA was amplified for 14 cycles to generate 5ʹ Gene Expression library. The Bioanalyzer (Agilent BioAnalyzer High Sensitivity Kit) was used for quality control and quantification of amplified cDNA. Finally, the cDNA library products were sequenced on Illumina Novaseq 6000 platform for single cell transcriptome libraries, with the following run parameters: read 1, 26 cycles; read 2, 98 cycles; index, 1–8 cycles. A median sequencing depth of 50,000 reads/cell was targeted for samples.

**scRNA-seq data processing and analysis**

The scRNA-seq data were processed using Cell Ranger (version 6.1.2, 10x Genomics). Reads were aligned to the human reference sequence GRCh38 with STAR alignment software. For quality control, we removed genes with detected expression in less than 0.1% of all the cells, and cells with less than 500 detected genes. Additionally, cells with the percentage of mitochondrial genes (>15%) and identified as doublets were also excluded. Finally, a total of 14406 cells were included for further analysis based on quality control metrics. After the identification of highly variable genes, the first 10 PCs (resolution=0.5) were applied for t-distributed stochastic neighborhood embedding (t-SNE) and Uniform manifold approximation and projection (UMAP) analysis. We used the Seurat package (version 4.1.0) in R (version 4.1.2) to perform data filtering, normalization, principle component analysis (PCA), t-SNE and UMAP after quality control. The Seurat function “FindAllMarkers” and Wilcoxon test were used to identify marker genes for each cluster.

***RNA sequencing***

The procedures of RNA sequencing (n=20), quality control, and normalization of data were performed in Shanghai Rightongene Biotechnology Co. Ltd (Shanghai, China), generally including the following steps: (1) Total RNA was extracted from the FFPE lymph node tissue section of AITL (10 μm thick) using RNeasy FFPE kit (Qiagen) according to the manufacturer’s instructions. (2) RNA quantity was assessed on Nanodrop, and the integrity of total RNA was estimated by RNA 6000 Nano Kit on Aligent 2100 Bioanalyzer. Then the RNA was used for the Library construction of cDNA (Illumina). The average size of the cDNA libraries was approximately 200bp (excluding the adapters). (3) The cDNA libraries were sequenced with 150 base paired-ends on the Illumina Hi-SeqTM 4000 platform. Sequencing was done on Illumina Hiseq platform using 150 bp pair-end sequencing strategy. Libraries were sequenced on the Illumina HiSeq 4000 platform in 2×150-bp paired-end format. (4) Read-pairs were aligned to the human reference genome GRCh38 (Ensembl release 84) using STAR (version 2.5.2b). Gene expression values were normalized for downstream analysis. (5) The differentially expressed genes (DEGs) were obtained with R package “limma”(v3·38·3). Due to the limited sample size, the DEGs that met the FDR<0.1 and |LogFC|>1 criteria were identified.
